# FDA-Approved and Emerging Next Generation Predictive Biomarkers for Immune Checkpoint Inhibitors in Cancer Patients

**DOI:** 10.3389/fonc.2021.683419

**Published:** 2021-06-07

**Authors:** Ye Wang, Zhuang Tong, Wenhua Zhang, Weizhen Zhang, Anton Buzdin, Xiaofeng Mu, Qing Yan, Xiaowen Zhao, Hui-Hua Chang, Mark Duhon, Xin Zhou, Gexin Zhao, Hong Chen, Xinmin Li

**Affiliations:** ^1^ Clinical Laboratory, Qingdao Central Hospital, The Second Affiliated Hospital of Medical College of Qingdao University, Qingdao, China; ^2^ Liaoning Cancer Hospital and Institute, Cancer Hospital of China Medical University, Shenyang, China; ^3^ Department of Biology, University of California – Santa Cruz, Santa Cruz, CA, United States; ^4^ Shemyakin-Ovchinnikov Institute of Bioorganic Chemistry, Russian Academy of Sciences, Moscow, Russia; ^5^ Department of Biological and Medical Physics, Moscow Institute of Physics and Technology, Moscow, Russia; ^6^ World-Class Research Center “Digital Biodesign and Personalized Healthcare”, Sechenov First Moscow State Medical University, Moscow, Russia; ^7^ Academy of Medical Engineering and Translational Medicine, Tianjin University, Tianjin, China; ^8^ Department of Pathology & Laboratory Medicine, University of California, Los Angeles (UCLA) Technology Center for Genomics & Bioinformatics, Los Angeles, CA, United States; ^9^ Department of Medicine, Qiqihaer First Hospital, Qiqihar, China

**Keywords:** immune checkpoint inhibitors, predictive biomarkers, PD-1, TMB, FDA-approved biomarkers

## Abstract

A patient’s response to immune checkpoint inhibitors (ICIs) is a complex quantitative trait, and determined by multiple intrinsic and extrinsic factors. Three currently FDA-approved predictive biomarkers (progra1mmed cell death ligand-1 (PD-L1); microsatellite instability (MSI); tumor mutational burden (TMB)) are routinely used for patient selection for ICI response in clinical practice. Although clinical utility of these biomarkers has been demonstrated in ample clinical trials, many variables involved in using these biomarkers have poised serious challenges in daily practice. Furthermore, the predicted responders by these three biomarkers only have a small percentage of overlap, suggesting that each biomarker captures different contributing factors to ICI response. Optimized use of currently FDA-approved biomarkers and development of a new generation of predictive biomarkers are urgently needed. In this review, we will first discuss three widely used FDA-approved predictive biomarkers and their optimal use. Secondly, we will review four novel gene signature biomarkers: T-cell inflamed gene expression profile (GEP), T-cell dysfunction and exclusion gene signature (TIDE), melanocytic plasticity signature (MPS) and B-cell focused gene signature. The GEP and TIDE have shown better predictive performance than PD-L1, and PD-L1 or TMB, respectively. The MPS is superior to PD-L1, TMB, and TIDE. The B-cell focused gene signature represents a previously unexplored predictive biomarker to ICI response. Thirdly, we will highlight two combined predictive biomarkers: TMB+GEP and MPS+TIDE. These integrated biomarkers showed improved predictive outcomes compared to a single predictor. Finally, we will present a potential nucleic acid biomarker signature, allowing DNA and RNA biomarkers to be analyzed in one assay. This comprehensive signature could represent a future direction of developing robust predictive biomarkers, particularly for the cold tumors, for ICI response.

## Introduction

Immunotherapy has changed the treatment landscape of many different cancer types in recent years. As opposed to chemotherapy and targeted therapy, which directly target tumor cells, immunotherapy stimulates a patient’s immune response or enhances a patient’s ability to fight against tumor cells. There are several different forms of immunotherapy used clinically, including cytokines, antibodies, vaccines, and immune checkpoint inhibitors (ICIs). Among those, ICIs are the most widely investigated and clinically used in the treatment of tumors.

ICIs target immune checkpoint regulators such as cytotoxic T-lymphocyte associated protein 4 (CTLA4), programmed cell death-1 (PD-1), or programmed death ligand 1 (PD-L1). Since the FDA approval of CTLA-4 inhibitor (ipilimumab) in 2011, the FDA has approved six more ICIs (1). Of those, three are PD-1 inhibitors (nivolumab, pembrolizumab and cemiplimab), and three are PD-L1 inhibitors (atezolizumab, avelumab, and durvalumab). These ICIs are widely utilized in around 15 tumor types (2) by oncologists in their daily practice and have shown remarkable efficacy.

However, ICI treatments are only effective in approximately 20% to 30% of cancer patients whose tumors are generally hot tumors with a high degree of T cell infiltration and high immune checkpoint expression (3). The majority of patients have no response or are resistant to the treatment, which is largely associated with cold tumors with few or absence of T cells, low tumor mutational burden, and poor antigen presentation (3). Furthermore, the efficacy varies among different tumor types, which further complicates treatment strategy. Given the expensive nature of immunotherapy, how to efficiently identify and select potential responders has become a clinical challenge to the effective use of ICIs. There is an urgent need to develop and validate more accurate biomarkers to assist in patient selection for ICI treatment.

Several different forms of predictive biomarkers have been developed for optimized use of immunotherapy, including positive predictive biomarkers to predict response to ICI, negative predictive biomarkers to predict resistance to ICI (4, 5), and side effect biomarkers to predict immune-related toxicity (6). Of those, the most validated and clinically used biomarkers for ICI responses are three FDA-approved positive predictive biomarkers: programmed death-ligand 1 (PD-L1), microsatellite instability/defective mismatch repair (MSI/dMMR), and tumor mutational burden (TMB). These three biomarkers have been reviewed extensively in the literature. For the most recent review, the readers can refer to Alessandro Rizzo et al.’s article in biliary tract cancer (7). Here, we do not intend to further review those biomarkers in general. Instead, we will focus on the challenges and solutions for effective use of these FDA-approved biomarkers.

The use of these three FDA-approved biomarkers has played a significant role in assisting appropriate selection of patients for ICI treatment. However, PD-L1, MSI/dMMR, and TMB each have different assays suitable to distinct tumor types and unique limitations. There is a lack of well-defined best practices to implement these biomarkers. In this article, we will review these three widely used biomarkers in clinical practice and discuss their strengths and weaknesses with the aim to standardize and optimize methodology. We will also review four promising gene signature biomarkers and two combinational gene signature biomarkers with an aim to explore more effective and accurate biomarkers suitable for larger tumor patient population, including immunologically cold tumors. These new forms of biomarkers are emerging and have shown impressive predictive power for ICIs. Finally, we will explore a comprehensive nucleic acid biomarker for future direction.

## Three FDA Approved Predictive Biomarkers

### PD-L1

#### FDA Approval and Rationale

PD-L1 was the first FDA-approved predictive biomarker for non-small-cell lung cancer (NSCLC) in 2015. Since then, the FDA has proved PD-L1 as a companion or complementary diagnostic test for six additional tumor types (gastric or gastroesophageal junction adenocarcinoma, cervical cancer, urothelial carcinoma, head and neck squamous cell carcinoma (HNSCC), esophageal squamous cell carcinoma (ESCC), and triple-negative breast carcinoma (TNBC)). Today, PD-L1 is the most investigated and clinically used predictive biomarker for ICIs.

PD-1 and PD-L1 belong to the family of immune checkpoint proteins. Their interaction plays a key role in regulating the immune system to ensure that it is activated only at the appropriate time to minimize excessive inflammation and autoimmune reactions. PD-L1 is expressed on a variety of normal and immune cells such as dendritic cells, activated T and B lymphocytes, and macrophages. However, tumor cells have also adopted this PD-1/PD-L1 interaction mechanism through expressing PD-L1 on the tumor cell surface. Binding of tumor PD-L1 to PD-1 on T cells results in attenuation or inhibition of T cell activity, which helps tumor cells escape from immune surveillance (8).

Blocking the PD-L1 and PD-1 interaction enables the reactivation of T cells and enhancement of T cell activity to fight tumor cells. Since the number of tumor cells that express PD-L1 largely affects its ability to suppress immunogenicity and further determine the effectiveness of PD-L1 and PD-1 blockage by ICI, the expression of PD-L1 on tumor cells is a predictive biomarker for ICI therapy.

#### Different Test Methods and Challenges

Four FDA-approved IHC testing methods are available today for measuring PD-L1 expression (**Table 1**). These methods use different antibodies, different scoring systems, different PD-L1 expression thresholds, and different types of cells expressing PD-L1. These variables among four methods are reflected in FDA approvals across seven different tumor types (**Table 2**).

**Table 1:** Variables for FDA Approved PD-L1 Test.

**Testing Method**	PD-L1 IHC 22C3 pharmaDx PD-L1 IHC 28–8 pharmaDx assay PD-L1 IHC SP 142 PD-L1 IHC SP263
**Antibody**	Monoclonal mouse anti PD-L1 Clone 22C3 Monoclonal rabbit anti PD-L1 Clone 28-8 Monoclonal rabbit anti PD-L1 Clone SP26 Monoclonal rabbit anti PD-L1 Clone SP142
**Scoring System**	TPS - Tumor Proportion Score, which is the percentage of viable tumor cells showing partial or complete membrane staining at any intensity CPS- Combined Positive Score, which is the number of PD-L1 staining cells (tumor cells, lymphocytes, macrophages) divided by the total number of viable tumor cells, multiplied by 100 %IC - The proportion of tumor area occupied by PD-L1 expressing tumor-infiltrating immune cells of any intensity
**PD-L1 Expression Threshold**	>=1% >=5% >=10% >=50%
**Type of Cells Expressing PD-L1**	Tumor cells for NSCLC Tumor-infiltrating immune cells for the triple-negative breast cancer Tumor and immune cells for the cervical cancer

**Table 2:** Key Parameters for Use of FDA Approved PD-L1 Testing for Immune Checkpoint Inhibitors.

Test Name	PMA#	Tumor Type	ICI	Approval Year	Scoring System	PD-L1-Threshold	PD-L1 Staining
PD-L1 IHC 22C3 pharmDx	P150013	NSCLC	Pembrolizumab	2015	TPS	>=50%	tumor cells
PD-L1 IHC 22C3 pharmDx	P150013/S006	gastric or GEJ adenocarcinoma	Pembrolizumab	2017	CPS	>=1	tumor cells, lymphocytes, macrophages
PD-L1 IHC 22C3 pharmDx	P150013/S009	Cervical Cancer	Pembrolizumab	2018	CPS	>=1	tumor cells, lymphocytes, macrophages
PD-L1 IHC 22C3 pharmDx	P150013/S011	urothelial carcinoma	Pembrolizumab	2018	CPS	>=10	tumor cells, lymphocytes, macrophages
PD-L1 IHC 22C3 pharmDx	P150013/S014	head and neck squamous cell carcinoma	Pembrolizumab	2019	CPS	>=1	tumor cells, lymphocytes, macrophages
PD-L1 IHC 22C3 pharmDx	P150013/S016	esophageal squamous cell carcinoma	Pembrolizumab	2019	CPS	>=10	tumor cells, lymphocytes, macrophages
VENTANA PD-L1(SP142) Assay	P160002/S006	urothelial carcinoma/NSCLC	atezolizumab	2018	IC%/IC% or TPS	>=5%/>=10% or >=50%	tumor area/tumor area, tumor ells
VENTANA PD-L1(SP142) Assay	P160002/S009	Triple-Negative Breast Carcinoma	atezolizumab	2019	IC%	>=1%	tumor area
VENTANA PD-L1(SP142) Assay	P160002/S012	NSCLC	atezolizumab	2020	IC%/TPS	>=10%/>=50%	tumor area Tumor cells
PD-L1 IHC 28-8 pharmDx	P150025/S013	NSCLC/SCCHN/UC	Nivolumab in combination with ipilimumab	2020	TPS	>=1%	tumor cells
**PD-L1 IHC SP263**	P160046	urothelial carcinoma	Durvalumab	2017	TPS/ICP/IC+	>=25%/ICP > 1% and IC+ >=25%/ICP = 1% and IC+ = 100%.	tumor cells Immune cells

These variabilities have posed practical challenges for clinicians and pathologists in daily practice. There is often confusion surrounding the different FDA-approved parameters in the tumor type and specific ICI administered. Consequently, although most widely used, PD-L1 has poor diagnostic accuracy overall, with a particularly low negative predictive value. For example, up to 20% of patients have PD-L1 negative tumors were reported to benefit from PD-1/PD-L1 antibodies (9). In addition to testing variables that contribute to low diagnostic accuracy, PD-L1 itself, as a predictive biomarker, has a relatively low predictability. Davis and Patel (2) analyzed 45 PD-L1 FDA approvals from 2011 to April 2019, and found that PD-L1 was only predictive in 28.9% of the approvals. Furthermore, PD-L1 expression is temporally and spatially regulated (10) and can be altered with prior therapeutic treatment (11). The combination of these factors limits PD-L1’s predictability in certain circumstances.

#### Future Directions

Although PD-L1 testing has low diagnostic accuracy overall, it has value for certain tumor types and remains the most widely used predictive biomarker in current clinical practice. A recent systematic review and meta-analysis showed that PD-L1 can effectively predict survival benefit in the patients with metastatic urothelial carcinoma (12), and soluble forms of PD-L1 and PD-1 in plasma samples can also predict sunitinib efficacy in patients with metastatic clear cell renal cell carcinoma (13). To improve clinical utility of PD-L1, future efforts should be directed to the following three areas:

a) Making effort to standardize future assay in clinical trials. Current variability of four PD-L1 assays is largely attributed to the initial clinical trials that had evaluated different PD-1/PD-L1 antibodies, used different scoring criteria and cut-offs for PD-L1, and stained different cell types. The organizations that design future clinical trials should consider possible standardization for the areas that can be potentially standardized in the planning stage.
b) Exploring standardization of currently-approved assays for clinical practice. For the currently-approved four commercial PD-L1 assays, we should explore possible standardization. A recent multi-center study compared the performance of 4 PD-L1 assays in lung cancer (14). They found that 22C3 for pembrolizumab, 28-8 for nivolumab, and SP263 for durvalumab are comparable to each other in the staining of tumor tissue. This result opens the possibility of using specific tests interchangeably. Among 11 FDA-approved PD-L1 linked companion diagnostic tests for seven tumor types (https://www.fda.gov/medical-devices/vitro-diagnostics/list-cleared-or-approved-companion-diagnostic-devices-vitro-and-imaging-tools), six tests for six tumor types used the PD-L1 IHC 22C3 pharmDx assay, six tests for five tumor types used tumor cells and immune cells for PD-L1 staining, and five tests for five tumor types used Combined Positive Score (CPS) as the scoring system. CPS is the number of PD-L1 staining cells (tumor cells, lymphocytes, and macrophages) divided by the total number of viable tumor cells, multiplied by 100. Therefore, the PD-L1 IHC 22C3 pharmDx assay, tumor cells, and immune cells for PD-L1 staining and the CPS scoring system could be considered as bases for future standardization.
c) Standardizing routinely used PD-L1 test may face a practical challenge and will take time. Currently, the most reliable and effective approach is to follow FDA-approved parameters for PD-L1 assays in seven tumor types. Pathologists and oncologists should use specific ICIs, scoring systems, stained cells, thresholds, assay platforms, and tumor types according to the approved PD-L1 test, and must be cautious in using ICIs beyond the approved assays.

### MSI/dMMR

#### FDA Approval and Rationale

MSI/dMMR was the second FDA-approved predictive biomarker for the pembrolizumab treatment of adult and pediatric patients with unresectable or metastatic solid tumors in 2017. The approval of pembrolizumab for MSI-H (MSI-high)/dMMR cancer treatment was based on the evidence of efficacy (ORR of 39.6%, complete response rate of 7%, and duration of response of six months or longer in 78% of responding patients) from five clinical trials (15). This approval represents the first drug that has been approved for solid tumors in general based on a common biomarker rather than for a specific tumor type (e.g. PD-L1).

Tumors with a defective DNA mismatch repair (dMMR) system accumulate thousands of mutations across the genome. Since short tandem repeats are particularly prone to mismatch errors, dMMR-induced hypermutations are most frequently located in microsatellite regions (1–6 nucleotides short stretches of DNA). This condition is defined as microsatellite instability (MSI). MSI results from and is a marker of dMMR.

Tumors with dMMR will also have more mutations in non-MSI regions throughout the genome and expectedly have more neoantigens compared to those with intact MMR. This assumption has been demonstrated by experimental data. Le et al. (16) reported that an average of 1782 mutations were present in colorectal cancers with dMMR compared with 73 mutations in the same tumors with intact MMR; consistently, 578 and 21 predicted neoantigens were found, respectively. The increased neoantigens in dMMR tumors are positively associated with overall lymphocytic infiltration, tumor-infiltrating lymphocytes, T helper 1 cells, and memory T cells (17, 18), which will render more effective antitumor immune response and a higher likelihood of response to immunotherapy. Thus, MSI/dMMR is a rational predictive biomarker for the treatment response to ICIs targeting PD-1, PD-L1, and CTLA-4 checkpoint receptor in such tumors.

#### Different Test Methods and Challenges

The FDA has approved pembrolizumab to be used in advanced MSI-H/dMMR solid tumors, but has not specified which assay should be used to measure MSI-H/dMMR. There are three different assays available for determining MSI-H/dMMR status in clinical practice: IHC for detecting dMMR, and PCR and NGS for detecting MSI-H (19–21).

IHC test for determining dMMR involves four proteins: MLH1, MSH2, MSH6, and PMS2. Loss of expression of one or more MMR proteins is considered as dMMR. MLH1 and MSH2 are obligatory proteins, and PMS2 and MSH6 are secondary proteins. PMS2 and MSH6 can form a heterodimer only with MLH1 and MSH2, respectively, while MLH1 and MHS2 can form heterodimers with other MMR proteins in addition to PMS2 and MSH6, respectively. The mutations in obligatory proteins result in functional loss of both obligatory and secondary binding partners, but the reverse is not true because secondary proteins can be substituted in the heterodimer by other MMR proteins. Consequently, antibodies against the secondary proteins detect mutations in both obligatory and secondary proteins, but antibodies for obligatory proteins alone do not detect mutations in PMS2 or MSH6 abnormalities. For this reason, some of the IHC assays only test PMS2 and MSH6.

IHC is a simple, cost-effective and widely available laboratory test that can be easily performed in all hospitals, clinics, and testing labs. The downside of IHC is a relatively low analytic sensitivity and accuracy due to technical or biological reasons. Technical reasons resulting in false negative staining can include pre-analytical issues, such as tissue fixation (22). Biologically, missense mutations in any MMR gene that can result in functional inactivation of a protein without affecting its antigenicity and expression levels (23).

PCR test is the second established method for determining MSI-H. Several PCR panels have been proposed, but two are most widely used in clinical practice: (i) a panel with two mononucleotide (BAT-25 and BAT-26) and three dinucleotide (D5S346, D2S123 and D17S250) repeats, which was proposed in 1997 by an international consensus group, also known as the Bethesda panel (24). Both tumor and paired normal tissue are required for using this panel; (ii) a panel with five poly-A mononucleotide repeats (BAT-25, BAT-26, NR-21, NR-24, NR-27). This five poly-A panel has a higher sensitivity and specificity compared to the Bethesda panel (25) and also does not need corresponding normal tissue for the test. If two of these five biomarkers in either panel lose stability, the tumor is diagnosed as having MSI-H. Recently, Thermo Fisher released a new TrueMark MSI Assay with a panel of 13 microsatellite biomarkers. In addition to expanded content from the five poly-A panel discussed above, this panel has a faster and simpler workflow, requires only 2ng FFPE tumor DNA and does not require the use of a tumor-normal match.

Since MSI testing by PCR is based on a specific and limited number of microsatellites analyses, the test cannot capture full MSI profiles and thus misses around 0.3% to 10% of cases (26). Furthermore, although MSI can be present in almost all solid tumor types, its prevalence and type of MSI are widely variable across the different tumor types. Several major cancer types, like NSCLC, breast cancer and prostate cancer have only 1-2% prevalence while other cancer types, such as melanoma and kidney cancer, have no data available (27, 28). The majority of clinical data for predictive ability for ICIs were largely from CRC. These factors limit its use as an effective and reliable predictive biomarker for ICIs in a broad scale, despite being approved for all solid tumors.

NGS-based MSI-H/dMMR testing is a relatively new assay and can overcome the limitations of MSI testing by PCR to a certain degree. NGS test uses either cancer gene panels or whole exome sequencing. For cancer gene panels, the number of genes varies from focused cancer gene panes with around 500 genes to comprehensive cancer exomes with >5000 genes (29). A bioinformatics method, MSIsensor, has also been developed to predict MSI status using whole exome data (30). The MSIsensor prediction showed 100% agreement with gold standard methods of IHC and PCR for MSI testing in 130 CRC patients.

The main advantage of NGS is its ability to evaluate a large number and different types of microsatellites including two- to six-base repeats, and to discover additional microsatellites with better predictive power. As opposed to PD-L1 and MSI testing, which are primarily suitable for metastatic colorectal cancer and other cancers belonging to the spectrum of Lynch syndrome, NGS method can be used for all tumor types, including non-Lynch syndrome rare cancers for multiple ICIs. Because NGS is the primary method to evaluate TMB, which will be discussed later, another advantage of NGS-based MSI-H/dMMR testing is the ability to integrate MSI with TMB data for the prediction of ICIs. The main challenges of NGS testing are its high cost, technical demands and lack of wide availability. Once these hurdles are overcome, NGS-based MSI testing will be a more accurate and sensitive assay than PCR or IHC for determining MSI status (21).

#### Future Directions

IHC-dMMR, PCR-MSI-H, and NGS-MSI-H each have strengths and weaknesses (**Table 3**). Although agreement has been found among the three methods, especially in CRC, differences exist across cancer types. The FDA has granted approval for the use of Pembrolizumab, nivolumab, and nivolumab–ipilimumab combination in metastatic solid cancers with MSI-H or dMMR, but did not specify which assay should be used to measure MSI status. A clear guideline is needed to help pathologists make informed decisions about which method to use in a given clinical situation. The CAP and three collaborating societies are developing a clinical guideline for testing MSI in patients with a range of cancer types. The groups opened the public comment period for the guideline in February, which ended on March 13, 2020. Formal guidelines are expected to be released soon. The European Society for Medical Oncology (ESMO) has already published its recommendations as of 2020 (28). Taken together, three general considerations can improve effective utilization of these assays:

a) The first and most important consideration is the prevalence of MSI in different tumor types. Although MSI-H can be present in almost all solid tumor types, its prevalence is widely variable across the different tumor types. MSI testing should be performed using IHC, PCR, or NGS method for the tumor types with high frequency of MSI, generally belonging to the spectrum of Lynch syndrome, including colorectal cancer (31), endometrial cancer (32), gastric cancer (33), ovarian cancer (34), and small Intestinal cancer (35). For other tumor types that do not belong to the spectrum of Lynch syndrome with low prevalence of MSI or no MSI data available on the reliability of IHC and the PCR method, such as NSCLC, breast cancer, melanoma, and kidney cancer, NGS-MSI should be considered because the NGS method can scan all types of MSI and also couple analyses of MSI with TMB.
b) The second consideration is the order of testing methods. In consideration of availability, cost and ease of testing, ESMO recommends IHC-dMMR as the first choice, and then PCR-MSI when IHC results are indeterminate. However, previous studies showed that the expression of MMR proteins, commonly MSH6, can change after neoadjuvant therapy (36, 37) and that dMMR tumors are more common in early-stage disease of different cancer types (defined as stage <IV) compared to advanced and metastatic settings (38). Given these two variables, the PCR-MSI should be a preferred testing method over IHC after neoadjuvant therapy or in advanced tumors. The last choice is NGS-MSI. The primary reason for recommending NGS-MSI last is due to the assay complexity, high cost and lower accessibility. Another complication of the NGS method is the determination of the appropriate threshold for calling MSI-H. Different NGS panels with different numbers of genes and different tumor types with different MSI frequency each impact threshold determination. It is practically difficult to reach a consensus threshold, which needs to be determined empirically and validated clinically for a specific NGS panel.
c) The third consideration is panel selection. For IHC-dMMR, antibodies for four MMR proteins (MLH1, MSH2, PMS2 and MSH6) should be used instead of MSH6 and PMS2 only. The mutations in MLH1 and MSH2 lead to loss of MLH1 and PMS2, and MSH2 and MSH6, respectively. However, there are isolated losses of PMS2, MSH2, or MSH6, which supports the notion of using all four antibodies to improve testing certainty and accuracy. For PCR-MSI, a panel with five poly-A mononucleotide repeats (BAT-25, BAT-26, NR-21, NR-24, NR-27) is recommended over a panel with two mononucleotides (BAT-25 and BAT-26) and three dinucleotides (D5S346, D2S123 and D17S250) for higher sensitivity and specificity. For NGS-MSI, the number of genes in the panel should be at least >300. A panel of 2000-5000 genes may be a good compromise between cost and coverage.

**Table 3:** Strengths, weaknesses and recommendations for three predictive MSI-H/dMMR biomarkers for ICI response.

Assays	Strengths	Weaknesses	Recommendations
**IHC for dMMR**	Simple Fast Cost-effective Widely available	Too many variables Hard to determine cut-off Relatively low analytic sensitivity and accuracy	First choice in general Use of all four antibodies Use for colorectal cancer and other spectrum of Lynch syndrome when suitable
**PCR for MSI-H**	Widely available Ease of use Accurate for colorectal cancer and other spectrum of Lynch syndrome	Capture partial MSI profiles Low prevalence in some tumor types	Use of five poly-A panel Use after neoadjuvant therapy or in advanced tumors
**NGS for MSI-H**	Capture full MSI profile Suitable for all tumor type More accurate and sensitive Simultaneous detection of other potential predictors	High cost Technical demands Lack of wide availability Need tumor-type specific cut-off	The last choice >300 genes in the panel Standardize technical parameters wherever possible

### TMB

#### FDA Approval and Rationale

TMB is a measure of the number of gene mutations in cancer cells and can be reported as the total number of nonsynonymous somatic mutations in the tumor exome (39) or per megabase DNA (40). TMB was recently approved for pembrolizumab for the treatment of adult and pediatric patients with unresectable or metastatic solid tumors in June 2020. Foundation One CDx assay (Foundation Medicine, Inc.) was also approved as a companion diagnostic test.

Several key factors can contribute to elevated TMB, including cigarette smoke, ultraviolet radiation, and defective damage response (DDR) genes (40). Among those factors, mutations in the DNA damage response (DDR) genes are particularly important, and emerging as independent predictors for ICI response. Teo et al. (41) observed that mutations in DDR genes are significantly associated with clinical benefit in patients receiving immunotherapy. Similar results were also reported in colorectal cancer (42), urothelial cancer (43), and serval other cancers (44). For a most recent review in this topic, please reference Minlin Jiang et al. (45). A high number of mutations in somatic exonic regions will lead to an increase in neoantigen production, some of which are immunogenic, and could then be recognized by T cells, resulting in improved antitumor immune responses. Consequently, patients with high TMB likely produce more intensified immune responses and are more sensitive to ICI treatments.

#### Different Test Methods and Challenges

There are 2 primary methods for evaluating TMB: WES and NGS panels. WES-TMB was first demonstrated to have an association with ICI response and proposed as a predictive marker for ICI by Snyder et al. (46) and Rizvi et al. (47), followed by many others (48, 49). These early WES-TMB studies count only nonsynonymous somatic mutations. TMB-H (TMB high) was called using different cutoffs varying from ≥7.4 in Esophagogastric cancer and ≥23.1 in NSCLC when the number of nonsynonymous somatic mutations was reported as per megabase DNA, and from ≥158 mutations in Advanced NSCLC to ≥248 mutations in advanced SCLC when whole tumor exome bases were counted. These different reporting formats and cutoff values complicate clinical practice. In addition, the clinical utility of WES-TMB was limited by high cost, long turn-around time, technical complication, and availability (40).

To address the WES-TMB limitations, researchers developed NGS panels with a sufficiently large number of cancer-targeted genes to predict TMB (50, 51). Early pioneer studies demonstrated that properly designed and sufficiently large NGS panels can accurately recapitulate WES-TMB and be effectively used as an independent predictor of ICI treatment. Further analyses provided additional evidence on reproducibility, repeatability, and the limit of detection compared with WES, and demonstrated good agreement between NGS panels-derived and WES-derived TMB data (52, 53). Importantly, these targeted NGS panels with fewer DNA bases and relatively simpler assays have improved utility in clinical settings.

The calculation of TMB has different methods, depending on the assay adopted. The WES-TMB assays typically consider nonsynonymous somatic mutations in the analysis, while NGS panels have generally taken a more comprehensive approach, such as FundationOne CDx, which includes synonymous and non-synonymous single-nucleotide variants (SNVs) for improved assay sensitivity (54), and insertions and deletions (indels) per area of coding genome sampled, but excludes known and likely oncogenic driver events and germline SNPs. There is currently no standard of TMB calculation. The TMB Harmonization Project is aimed to standardize TMB calculation and reporting (55–57).

There are two NGS panels commercially available that have been approved by regulatory bodies: (i) MSK-IMPACT with 468 cancer genes was cleared by the 510K pathway for mutation profiling in November 2017, and (ii) the FoundationOne CDx assay with 324 cancer genes was approved by the FDA as a companion diagnosis for the evaluation of TMB in 2020. These targeted gene panels can analyze and identify single nucleotide substitutions, indels, CNAs, and selected gene rearrangements, as well as genomic signatures including microsatellite instability (MSI) and loss of heterozygosity in a single assay.

Overall, TMB as a predictive marker for ICI treatment is more technically challenging than PD-1 and MSI. Many variable factors can impact TMB estimation and output, including tumor type (different tumor types biologically have different TMB (39)), tissue type (FFPE tissue will artificially have more mutations than fresh frozen tissue), sequencing parameters (NGS panel content, size and sequencing depth, bioinformatics pipeline), and the reporting cutoff (55). The wide variation in TMB estimation and reporting methods across studies have limited effective adoption of TMB and stressed the need to standardize assays for determining TMB.

#### Future Directions

TMB-H is generally predictive of response to multiple forms of ICIs, but the predictive ability can vary across tumor types and mutation types. Since the affinity of neoantigen binding to MHC1 and T cell receptor recognition of neoantigen as foreign are two determinants of immune response, distinct qualities of neoantigens contribute to ICI response differently. Generally, the lack of similarity of neoantigen to self-antigens results in an increased ability to activate T-cells, and thus, predicts response to ICIs. For example, Merkel cell carcinoma (MCC), renal cell cancers (RCC), and mesothelioma all have higher response rates to ICIs than would be anticipated from their TMBs (58) due to the higher quality of antigens in these tumor types. Elevated antigen quality results from viral antigens (in MCC), a high number of indel mutations (in RCC), and complex chromosomal rearrangements (in mesothelioma) (59). Keeping these in mind, the below 3 points should be considered in TMB estimation and reporting:

a) A clinically validated, sufficiently large NGS panel is preferred over WES. In consideration of clinical utility (low cost, shorter turn-around time, use of smaller biopsy samples, higher assay sensitivity, lower technical complexity and bioinformatics demand), a standardized, commercially available NGS panel, such as FDA-approved FoundationOne CDx, is recommended for TMB determination. When FoundationOne CDx panel is used, ones should follow approved method for TMB calculation (Douglas B et al., 2016). The panel should be sufficiently large, including ≥300 targeted genes. These genes should be carefully selected by including the following: (i) other TMB-related marker genes, such as POLE whose mutations are associated with TMB-H in multiple solid tumor types like endometrial, CRC, gastric, melanoma, lung, and pediatric cancers (60–62), or BRAF and MET whose alterations are associated with longer duration of ICI treatment; (ii) other immunotherapy response-related genes, such as genes for MSI estimate, immune resistant gene, IDO1, and JAK (4, 5); (iii) multiple types of alterations, such as mutations, indels, amplifications, CNAs, and structure variations. Such a panel will be small enough for broader clinical application, but informative enough to allow performing multiomic analyses to provide a more comprehensive, complete, and robust patient biomarker profile for independent or joint ICI treatment decisions.
b) Weighted calculation for TMB score. Since different types of alterations have variable immunogenicity, one should not only focus on the number of mutations, but also consider the types of mutations when evaluating TMB. Generally, patients with frameshift indels, transversions, and clonal mutations are more immunogenic than those with nonsynonymous mutations (63), transitions (47), and branching or subclonal mutations (64), respectively. In calculating TMB status, the index may be expressed as a TMB score. The more immunogenic types of mutations should be preferably weighted. By using a TMB score, other factors that also affect TMB predictive value can also be considered, such as age (65).
c) Tumor-type specific reporting cutoffs. TMB estimation and reporting methods are widely variable in scientific publications. Like PD-L1, there is an urgent need to standardize current TMB assessment methods, which is essential for reliable use of TMB as a clinical biomarker for ICI response. However, among these variables, some technically related variations can be addressed by standardization, such as sequencing depth and gene panel, while others related to biological variations can be addressed according to biology. The variation in reporting cutoff is a typical example of biological variation. Some tumor types have naturally higher TMB than others (66), and thus require a different cutoff for reliable and reproducible ICI response prediction. For example, >16 mutations/Mb is appropriate for atezolizumab in urothelial carcinoma (67), while >23.1 mutations/Mb is needed for pembrolizumab in NSCLC (68). In fact, the TMB cutoff varied markedly across the top 20% of each cancer type (66), suggesting that it is unlikely to be able to use a universal cutoff. The optimal cutoff should be developed and validated in different tumor types.

## Non-FDA Approved Emerging Biomarkers

### Promising Mutation Predictive Biomarkers

#### Inactivation of PTEN

PTEN is ubiquitously expressed protein phosphatase that is one of the major human tumor suppressors (69). For example, it dephosphorylates PIP3 to PIP2 and thus inhibits PI3K/mTOR/Akt signaling axis (70) and serves as the potent regulator of DNA repair (71). Even a single-allele mutation of *PTEN* can irreversibly repress molecular functions of this gene, thus making a cell susceptible to carcinogenesis (72). Decreased expression of *PTEN* is also connected with the sensitivity to ICIs which can be mediated by lower infiltration of such cells by T-lymphocytes (73). In lung cancer, mutations of *PTEN* were shown to be associated with poor response to ICI therapy (74).

#### Mutations of POLE

POLE is a subunit of DNA polymerase epsilon that has polymerase and proofreading activities, and participates in both DNA replication and repair (75). Mutations in proofreading domain of *POLE* are present in 1-12% of all tumors (76, 77) and result in approximately two orders greater mutation rate, thus directly influencing TMB (78). Tumors with *POLE* mutation have more neoantigens and more infiltrating lymphocytes (79).

#### Linked Mutations of KRAS and STK11

Somatic activating mutations in 12 and 13 codons of *KRAS* can be detected in 5-35% of the patients in different cancer types (80) and most frequently are associated with poor survival prognosis (81). These mutations are statistically significantly linked with the mutations in the *STK11* gene (82) that encodes LKB1 kinase which phosphorylates and activates AMPK, a potent metabolic regulator (83) that controls mTOR signaling (84). In lung cancer, up to 30% of tumors may have mutated *STK11* (85), and presence of both *STK11* and *KRAS* mutations is a factor of poor survival prognosis (86). Inactivation of *STK11* is also a factor of more inert tumor microenvironment and lower expression of PD-L1 (87).

In lung cancer patients with mutant *KRAS*, ICI therapy showed lack of benefit, in contrast to the wild-type group of tumors (88). In *KRAS* mutant tumors, less patients responded on ICI therapy in a *STK11*-mutated subgroup compared to a *TP53*-mutated subgroup (7.4% versus 35.7%, respectively). The same was observed in clinical trials CheckMate057 (0% vs. 57,1%), CheckMate-012 (0% vs. 78%) (89), and GEMINI (0% vs. 53%) (87). This also reflected statistically lower time to progression (TTP) in patients having both mutations in *KRAS* and *STK11* genes compared to the tumor with only *KRAS* mutations (90).

In agreement with that, *KRAS*/*STK11* double mutant lung cancers showed worse survival compared to only *STK11* mutants: TTP of ~two months vs. five months, and overall survival of ~seven months vs 16 months (91). Bad prognosis of double mutant tumors was relatively independent on PD-L1 expression and was also true for the PD-L1-positive group of double mutants (90). Interestingly, these mutations can likely synergistically promote tumor infiltration by T cell suppressing neutrophils (92).

### Gene Signature Predictive Biomarkers

Three FDA-approved predictive biomarkers, PD-L1, MSI/dMMR, and TMB have played a critical role in guiding ICI treatment selection. However, each has its limitations. PD-L1 has limited positive and negative predictive values, MSI-H/dMMR has a low prevalence in many common metastatic cancers (<5%), and TMB is hindered by high cost and technical complications. Additionally, a wide range of response rates have been reported, such as patients with low TMB, absence of MSI or without PD-1/PD-L1 expression showing good response, or vice versa. This unpredictability clearly indicates that immunotherapy response is also driven by other biomarkers. The identification and validation of additional predictive biomarkers are needed. Recently, gene expression-based signatures have emerged as a new generation of predictive biomarkers for ICI response. Here, we will discuss four different gene signature biomarkers: T cell-inflamed gene expression profile (GEP), T cell dysfunction and exclusion gene signature (TIDE), melanocytic plasticity signature (MPS), and B cell-focused gene signature.

a) T cell-inflamed gene expression profile (GEP) is one of the early reported and clinically validated gene signatures for predicting response to pembrolizumab across multiple solid tumors (93). Through stepwise validation of several populations, an 18-gene pan-tumor signature was identified in 220 patients of nine different tumor types. This signature is represented by the genes related to IFN-γ signaling, cytotoxic effector molecules, antigen presentation, and T cell active cytokines, which is a common characteristic of a T cell-inflamed tumor microenvironment responsive to ICIs. Across multiple tumor types, data showed that responders have high level of signature gene expression (a T cell inflamed phenotype) while non-responders have low expression level across the signature genes (a non-T cell-inflamed phenotype). Its predictive value was demonstrated independently in a 96-patient population with head and neck squamous cell carcinoma. ROC analysis showed that the 10-gene signature has a larger area under the ROC curve than that of PD-L1, demonstrating that the T cell-inflamed multigene signature has a better predictive value compared to the commonly used single gene biomarker, PD-L1.
b) The second promising gene expression panel is the T cell dysfunction and exclusion gene signature, termed TIDE for Tumor Immune Dysfunction and Exclusion. Different from T cell-inflamed gene signature, which captures a favorable tumor environment for ICI response (a high level of gene expression in the panel is indicative of response), TIDE focuses on the loss of T cell functionality, which reflects an unfavorable tumor environment for ICI response (a high level of gene expression in the panel is indicative of non-response). TIDE was developed based on two key mechanisms of tumor immune evasion (94, 95): dysfunctional infiltrated T cells in the tumor, and prevention of T cell infiltration into the tumor. Using large data sets and computational modeling method, Peng Jiang et al. (96) identified gene signatures that underlie these two mechanisms of tumor immune escape separately and integratively.
  Using publicly available transcriptome profiles of non-treated tumors with patient survival outcomes, the authors first used Cox proportional hazard (Cox-PH) model to test the interaction of the expression of each gene in tumors with the level of T cell infiltration (defined as average gene expression of known regulators of T cell dysfunction) to influence patient survival. This systematic, statistical interaction test identified signature genes that affect T cell function and patient survival. The profiles of these genes are enriched by inflammatory and interferon response-related pathways and lack of pathways that promote T cell activation, reflective of T cell dysfunctional phenotype. Similarly, the authors used the expression profiles of three cell lines, MDSCs, TAMs, and CAFs that restrict T cell infiltration in tumors, to model T cell exclusion, and developed a gene signature of T cell exclusion. Finally, TIDE, an integrated signature, was developed to predict ICI response. ROC analysis showed that TIDE has better predictive performance than TMB and PD-L1 for both anti-PD1 and anti-CTLA4 therapies. In addition, a lower TIDE score is predictive of longer patient overall survival.
c) Melanocytic plasticity signature (MPS) was developed by studying four mouse immunocompetent melanoma models (M1-M4), which represent major subtypes of human cutaneous melanoma, and the diversity of clinical responses to ICIs. M1 and M2 mice had no response to anti-PD-L1 and sustained tumor growth, M3 mice had modest response and delayed tumor growth, and M4 had the best response and significantly longer survival time. By comparing RNA-seq data of ICI-resistant M1 and M2 and the sensitive M3 and M4, and subsequent evaluation of response prediction in the Van Allen dataset, Eva Pérez-Guijarro et al. (97) identified a 45-gene signature predictive to ICI response. Low MPS scores were significantly associated with responders. In the Van Allen dataset, 81% of responsive patients can be correctly predicted by using MPS score. Furthermore, the patients with low MPS had longer progression-free survival and overall survival.
  Further analyses showed that the 45-gene signature reflects the multipotency and differentiation of the melanocytic lineage. A high MPS score represents undifferentiation and multipotency, and a low MPS score indicates later stages of melanocytic differentiation. These data suggest multipotency and differentiation status of melanoma can predict ICI response, which represents a novel discovery. In a comparison of predictive performance among MPS, TIDE, TMB, and PD-L1, ROC analysis showed that MPS had the best ROC area under the curve (AUC) value followed by TIDE in the Van Allen and Hugo–Riaz data sets (97).
d) The B cell gene signature is a recently reported new biomarker for ICI response. Since current ICI treatments reinvigorate T cells against tumors, research of predictive biomarkers to ICI response in the past was largely focused on T cells. Several recent studies showed that the B cell rich immune cell population in tertiary lymphoid structures (TLS) of tumors is a critical discriminative feature of ICI responsiveness and patient overall survival (97–99). TLS are aggregates of immune cells and have been associated with increased patient survival in several cancer types. These recent studies further demonstrated that significantly enriched B cells localized in TLS, specifically switched memory B cells (99), are key predictors of ICI response. Helmink et al. also showed the presence of high diversity of B cell receptors in responders compared with non-responders. All these data demonstrated an active role of B cells and tertiary lymphoid structures in ICI response, and highlighted a possibility to develop predictive gene signatures for ICI response focused on B cells within TLS.

Cabrita et al. (98) constructed a TLS gene signature in metastatic melanoma. This signature is dominated by B cell-specific genes and is predictive of ICI response as well as patient overall survival. Cox regression analysis using several immune signatures across the four cohorts demonstrated that the TLS signature has the best predictive performance in the cohorts treated with anti-PD1. The predictive performance of TLS signature is independent of TMB. A similar B cell dominated gene signature was also developed in soft tissue sarcoma (100). Using the microenvironment cell populations (MCP-counter) method (101), the authors classified 608 tumors from different subtypes of soft-tissue sarcoma into five groups (A, B, C, D, and E) based on the composition of the tumor microenvironment. An immune-high group E was characterized by the high density of B cells and TLS. The key determinant of group E was the high expression of the B cell signature. Once again, the B cell signature was significantly associated with better ICI response and improved overall survival.

Of the above 4 gene signatures, the T cell-inflamed, GEP, and TIDE have superior predictive performance for ICI response compared to PD-L1, and PD-L1 or TMB, respectively. MPS has better predictive performance than PD-L1, TMB, and TIDE. The B cell focused gene signature is a new predictive biomarker, and its predictive value has yet to be thoroughly evaluated in relation to other established biomarkers. Based on currently available data, the gene expression-based signatures are generally more robust with enhanced predictive value compared to single gene or protein markers.

In addition, a proof of concept has been recently reported that next-generation expression signatures based on molecular pathway activation profiles (102, 103) using RNA sequencing data (104) can guide personalized ICI prescription in treatment refractory tumors (105).

### Combinational Predictive Biomarkers

**Figure 1:**
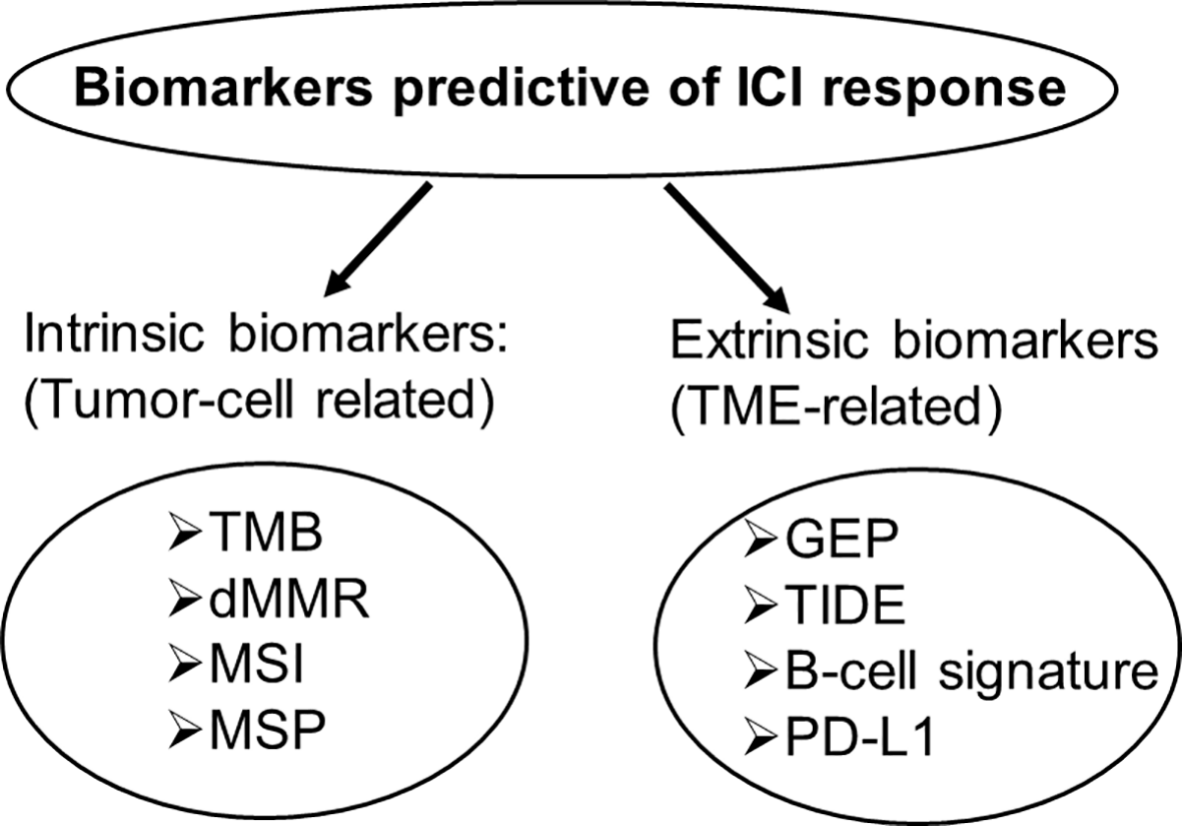
Intrinsic and extrinsic biomarkers predictive of ICI response. Intrinsic biomarkers are tumor cell-related, extrinsic biomarkers are tumor microenvironment-related.

Currently FDA-approved and recently developed gene signature biomarkers for ICI response fall into two broad categories: one category is related to tumor intrinsic factors, such as TMB, MSI and MPS, and the other category is related to tumor extrinsic factors, including PD-L1, T cell, and B cell gene signatures (**Figure 1**). These biomarkers have independent predictive values for ICI response, but predicted responders across those biomarkers have a generally low percentage of overlapping, particularly between these two categories. This lack of correlation, together with the demonstrated individual predictive values, indicates that these biomarkers measure different aspects of complex tumor immunobiology and capture unique features of ICI response phenotypes. This suggests that the combination of different biomarkers may provide complementary or additive effects and lead to an improved predictive performance. Here, we will review two combined predictive biomarkers, GEP+TMB and MPS+TIDE, to demonstrate their improved predictive performance.

1) **GEP+TMB**. T cell-inflamed GEP and TMB measure T cell activation (tumor microenvironment) and tumor antigenicity, respectively, representing unique aspects of tumor immunobiology. To understand the interplay between these two distinct categories of biomarkers, Cristescu et al. (106) explored the joint predictive response to pembrolizumab across 22 tumor types from four KEYNOTE clinical trials. The individual biomarker prediction was first performed, followed by classification of patients into four individual biomarker-defined response groups (GEP^lo^TMB^lo^, GEP^lo^TMB^hi^, GEP^hi^TMB^lo^, and GEP^hi^TMB^hi^) using predefined cutoffs for TMB and GEP. The highest response rate was observed among patients in the group of GEP^hi^TMB^hi^ in all four cohorts. No response was seen in the group of GEP^lo^TMB^lo^ in the pan-tumor and HNSCC cohorts, and intermediate response rate was observed in the group of either TMB^lo^GEP^hi^ or TMB^hi^GEP^lo^. These data demonstrated that the combination of two biomarkers offers higher sensitivity and greater predictive value compared to a single biomarker. Additionally, the patients in the GEP^hi^TMB^hi^ group had longer progression-free survival time.
  The joint utility of the GEP+TMB in predicting ICI response was further tested in TCGA database using 6384 patients of matched transcriptome and WES data across a wide range of tumor types. Consistent with the data derived from KEYNOTE clinical trials, the patients with GEP^hi^TMB^hi^ had the strongest response, and GEP^lo^TMB^lo^ group had no or poorest response to pembrolizumab, demonstrating that the improved response rate by joint prediction of GEP+TMB can be generalized across cancer types.
2) **MSP+TIDE**. As discussed above, MSP reflects cancer cell intrinsic factor (multipotency and differentiation), which is not associated with immune response, while TIDE represents extrinsic factor (immune phenotype) reflective of the tumor microenvironment. Given these different features, Guijarro et al. (97) hypothesized that the combination of MPS and TIDE scores will increase predictive value. Indeed, ROC analysis showed a noticeable improvement of the AUC values by MPS+TIDE compared to any of the single methods in the Van Allen cohort.

The improved ICI response by combining MPS and TIDE signatures translated into patient survival. Similar to the GEP+TMB analysis described above, melanoma patients were classified into three groups based on their MPS and TIDE scores. The low-MPS and low-TIDE group showed significantly longer PFS and OS, whereas the high-MPS and high-TIDE group exhibited the poorest survival in Kaplan–Meier analysis.

Altogether, the results demonstrated that combining cancer cell intrinsic and extrinsic factor-related gene signatures can improve the predictability of not only ICI response, but also patient survival. This integrated predictive biomarker may represent a future direction for additional biomarker discovery.

## Future Direction of Predictive Biomarker Discovery

**Figure 2:**
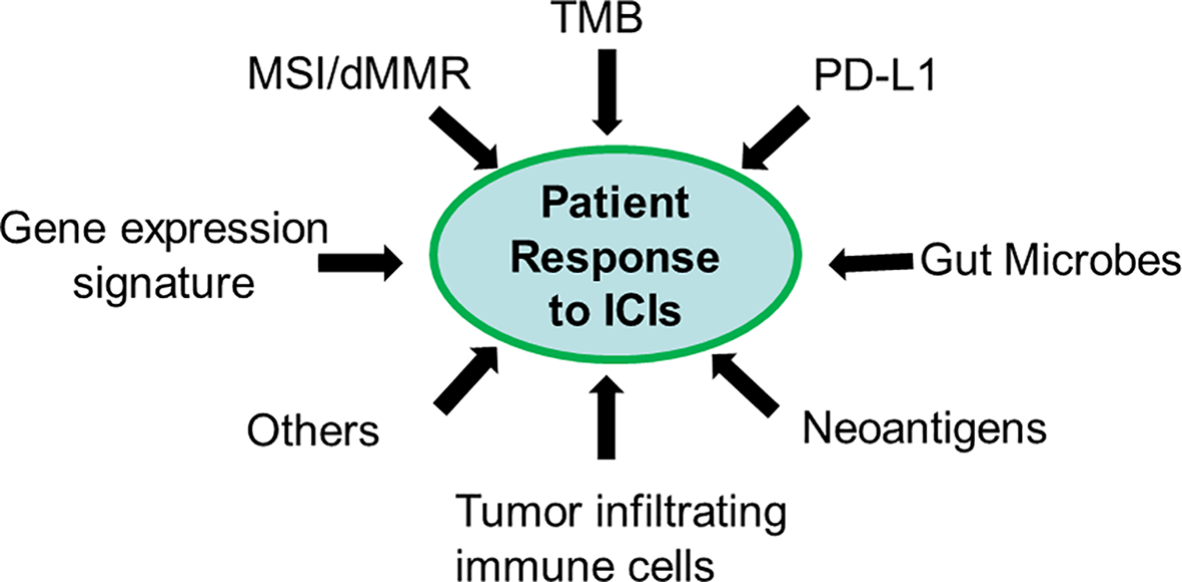
Patient response to ICIs is a quantitative trait. Each biomarker only captures a unique feature of the contributing factor (s).

The above analyses cover different predictive biomarkers from single to complex, DNA to RNA, and neoantigenic to TME-related. All data suggest that patient response to ICIs is a complex quantitative trait determined by multiple factors (**Figure 2**). Current biomarkers tend to capture a unique contributing factor of ICI response. Thus, a combination of biomarkers should offer improved predictive performance to ICI response. Because of ICI-related toxicities and the high cost of these agents, current predictive biomarkers with a highly variable response to ICIs cannot fully meet clinical need. There is an urgent need to develop a new generation of biomarkers that can reliably predict ICI response. Based on current knowledge and available data, an optimal ICI predictive biomarker is an integrated nucleic acid biomarker signature. This signature can combine information from different DNA and RNA biomarkers in one single assay to retrieve as many ICI response-related contributors as possible, from the upstream to downstream of immune response, from intrinsic to extrinsic factors, and from TME to neoantigenic aspects. A final combined index score will be used to predict ICI response, which will overcome potential conflicting results from different biomarkers in the same assay. Broadly, this integrated nucleic acid biomarker signature may include at least the following four categories:

1) TME-related RNA biomarker genes, including key T cell-inflamed signature genes, T cell dysfunction & exclusion signature genes, and B cell signature genes.
2) Tumor multipotency and differentiation related RNA biomarker genes, such as MSP signature genes.
3) Tumor neoantigenicity-related DNA biomarker genes including frequently mutated core cancer genes for TMB, DNA mismatch repair genes, and MSI panel.
4) Other high impact genes, such as *TGFB1* with a known role in promoting tumor immune escape and ICI resistance (107–109), *SOX10* with known function in promoting T cell-mediated tumor cell attacking (110, 111), *SERPINB9* with a demonstrated role in regulating ICI resistance, and *POLE/POLD1* with an established role in contributing to high TMB in some cancers (54). These high impact genes can come from both tumor cells and tumor infiltrating immune cells.

To ensure its clinical utility and economic feasibility, this integrated nucleic acid signature panel should be large enough to capture all key ICI responsive features and allow calculation of a reliable TMB, and small enough to be economically and technically feasible for broad application in daily clinical practice using next generation sequencing platforms. Because this integrated nucleic acid biomarker panel can comprehensively analyze DNA and RNA markers in one assay instead of two, it will have enhanced cost efficiency, reduced assay time, and require less biological material (total nucleic acids as input). This integrated assay includes multiple contributing factors to ICI response, and will likely be more predictive for immunologically cold tumors or advanced tumors.

## Author Contributions

YW and XL conceived and designed the project. AB and ZT wrote the part of FDA approved predictive biomarkers. WHZ and XM wrote the part of Promising mutation biomarkers. QY and XZha wrote the part of Gene Signature Biomarkers. H-HC and XZha wrote the part of Emerging Combined Predictive Biomarkers. YW, H-HC and XL wrote the part of Future direction of predictive biomarker discovery. WZZ and MD edited the manuscript. All authors contributed to the article and approved the submitted version.

## Funding

This study was supported by the National Natural Science Foundation of China (No. 81670822, 81800805), and Qingdao Key Research Project (No. 19-6-1-3-nsh). AB contribution was supported by the Ministry of Science and Higher Education of the Russian Federation within the framework of state support for the creation and development of World-Class Research Centers “Digital biodesign and personalized healthcare” No 075-15-2020-926.

## Conflict of Interest

The authors declare that the research was conducted in the absence of any commercial or financial relationships that could be construed as a potential conflict of interest.
